# Impact of Implementing the Critical Care Pain Observation Tool on Nurses' Performance in Assessing and Managing Pain in the Critically Ill Patients

**DOI:** 10.5005/jp-journals-10071-23146

**Published:** 2019-04

**Authors:** Mahnaz Modanloo, Afsaneh Mohsenpour, Hossein Rahmani, Shahram Moghaddam, Homeira Khoddam

**Affiliations:** 1,3,5 Nursing Research Center, Golestan University of Medical Sciences, Gorgan, Iran; 2 Critical Care Nursing, Nursing and Midwifery School, Golestan University of Medical Sciences, Gorgan, Iran; 4 Department of Anesthesiology, School of Medicine, Golestan University of Medical Sciences, Gorgan, Iran

**Keywords:** Analgesic administration, Critical care pain observation tool, Intensive care unit

## Abstract

**Background and aims:**

Pain management is one of the most important responsibilities of nurses in an intensive care unit (ICU). It is difficult to perform pain assessment appropriately in patients who are unable to report their pain. This study is aimed to determine the impact of implementing the critical care pain observation tool (CPOT) on the amount and frequency of analgesics' administration in ICUs.

**Materials and methods:**

This interventional study was conducted in 2014. Sixty nurses and 240 patients were studied. This study was carried out in three phases: first the data about amount and frequency of analgesic administration were extracted from patients' medical files. Then the CPOT was implemented into the nursing assessment process and finally, nurses' performance regarding the amount and frequency of analgesic administration was recorded. This data obtained before and after intervention were analyzed using chi-square and independent t-test *p* values less than 0.05 were considered significant.

**Results:**

In this interventional study, we found that there was no difference in the demography and cause of ICU admission before and after implementation of CPOT (age *p* = 0.937, gender *p* = 0.996, and the cause of admission *p* = 0.996). We found that after implementing the CPOT into the nursing assessment process, the amount of analgesics administered (7.95 ± 8.77 mg vs. 11.01 ± 11.04 mg, *p* = 0.018) and the frequency of administration (2.91 ± 1.38 vs. 4.16 ± 0.99, *p* <0.001) increased significantly. Moreover, there was a significant increase in the frequency of pain assessment per patient per day in nursing practice after implementation of CPOT as compared to the practice before (7.2 ± 2.48 vs. 1.03 ± 1.63, *p* <0.001). The mean pain scores before and after the intervention (5.5 ± 1.08 vs.2.2 ± 0.48) were also significantly different.

**Conclusion:**

Applying CPOT, as an objective mean of pain assessment, was effective in improving the performance of ICU nurses in assessment and management of patients' pain. It increased the amount and frequency of analgesic administration. We can recommend that COPT is a useful tool for assessment and management of pain in ICU patients and should be implemented in all ICUs.

**How to cite this article:**

Modanloo M, Mohsenpour A, *et al.* Impact of Implementing the Critical Care Pain Observation Tool on Nurses' Performance in Assessing and Managing Pain in the Critically Ill Patients. Indian J Crit Care Med 2019;23(4):165-169.

## INTRODUCTION

Pain is one of the most common complaints and the most severe stressors among intensive care unit (ICU) patients,^1,2^ which is mainly due to use of invasive devices, care providers' interventions such as endotracheal suction, dressing change, and position change.^1,3^ According to studies, 71% of patients in the ICUs recall their painful experience after recovering from their illness^4^ and many also claim that during this period their pain had not been truly relieved.^5^

Inadequate pain relief may lead to hemodynamic consequences, with increased workload of heart and higher risk of ischemic heart disease, pulmonary complications such as atelectasis, suppression of the immune system, and increased duration of hospitalization and mortality rate in patients.^6^

Pain management of ICU patients is a major healthcare concern.^7^ By using the proper approach to assess and manage the pain, we can prevent the problems mentioned above.^8^

Obviously, the patient's self-reporting is the most reliable indicator of pain assessment.^5,9^ In critical care units, many factors make verbal communication difficult for patients; such as intubation, low level of consciousness, hypnotics or sedative drug administration, endotracheal intubation, and mechanical ventilation.^10–11^

Pain is a subjective experience and there is no way to objectively measure it. Accordingly, accurate pain measurement depends on the patient's overt communication, both verbal and behavioral. Precise estimation and appropriate intervention for pain management pose a communication-related challenge between patients and healthcare providers.^12^ While patients are not able to communicate verbally and express their pain, observable indicators are the best and unique indices for pain assessment.^10,13–14^

Behavioral pain scales such as CPOT have been suggested by experts, and CPOT has been validated for use in adult patients in the ICU.^9^ The American Society for Pain Management Nursing recommends its use in all ICU patients.^7,8^ This tool has been suggested to be used for assessing pain in adult patients with and without endotracheal tube; who are not able to communicate verbally. Use of this tool leads to a precise estimation of pain severity and consequently choosing an appropriate intervention for pain management.^15^

Accurate pain assessment is required to ensure optimal pain management.^16,17^ Research finding reveals that caregivers frequently underestimate or overestimate pain intensity in more than 50% of their patients.^18^ However, a study has demonstrated that nurses often use invalid and biased methods to assess pain in patients^19^ and therefore, the majority of nurses (35-55%) underestimate the pain intensity.^20^ Furthermore, studies have shown that 64% of ICU patients, before and during painful medical care, do not receive care in order to relieve pain.^20^ Inadequate pain relief causes restlessness, myocardial ischemia, and lack of coordination with mechanical ventilation.^21^ On the other hand, the overuse of analgesics can also result in hypotension, respiratory depression, prolongation of mechanical ventilation, prolonged hospitalization, and respiratory infection associated with mechanical ventilation in the ICU.^6^

Studies have shown that training nurses on the method of pain assessment and relief alone, is not enough to alter their performance in patients' pain management, but it is necessary to have guidelines and implement observation tools to assess pain and pain management nursing practices.^19^ The purpose of this study was to determine the impact of implementing the CPOT on the amount and frequency of analgesics' administration in ICU patients.

## MATERIALS AND METHODS

This prospective interventional study with the before-and-after design was conducted in 2014. This study was undertaken to determine the effect of pain management program on nurses' performance and patients' clinical outcomes in ICU. The educational program consisted of two components-implementation of suggested objective tool, CPOT, as a part of the assessment process, and pain management.

The sample consisted of all ICU nurses (n = 60) and 240 eligible patients. The eligible patients were intubated and had a low level of consciousness (GCS 5-10). They did not have neuromuscular disorders, delirium, and addiction to alcohol or drugs; and were not receiving continuous infusions of hypnotics, painkillers, and neuromuscular-blocking drugs. Four eligible patients were chosen randomly for each nurse who took care of them, two patients before and two patients after implementation of the CPOT.

The instruments were organized to gather three types of data-nurses' information, patients' general and medical information, and data related to pain assessment and analgesics administration. In the first part, demographic variable of nurses including age and gender; and in second part, age, gender, and diagnosis of patients were collected; and in last part, the audit chart which was created for this study, was used to collect any information on pain assessment and relief such as amount and frequency of analgesics' administration, and some physiological parameters (systolic and diastolic blood pressures, heart rate, respiratory rate, and oxygen saturation of arterial blood). The CPOT was used to measure pain score in last part. CPOT includes four sections. Each section includes a specific behavioral indicator: facial expression, body movement, muscle tension, and compliance with the ventilator for mechanically ventilated patients or extubated patients (Table 1).^7^ Each domain is scored from 0 to 2, and the total score can range from 0 to 8, a score >2 is interpreted as the presence of pain.^8^

This study was carried out in 5th of Azar Medical Center after approval of Ethics Committee of Golestan University of Medical Sciences (code: 364392121122). This implementation study included three phases. In preimplementation phase, data were extracted from medical records of 120 patients during the first 24 hours of admission in the ICU. In the implementation phase, all ICU nurses (n = 60) attended a one-day educational workshop, in which they were trained to document patients' pain management step by step, by focusing on CPOT. The CPOT and its instructions were attached in each ICU unit and included in ICU flow sheet. In the post-implementation phase, data were extracted from 120 ICU patients' medical files once more, which were matching the age and gender of patients in the preimplementation phase.

Data were analyzed using SPSS version 16. Descriptive statistics were used to describe patients' and nurses' demographic characteristics and Chi-square and independent t-test were used to analyze data. The level of statistical significance was considered at less than 0.05.

## RESULTS

According to the findings, the majority of nurses participating in this study were female (88.3%), married (68.3%), and with a bachelor's degree (95%). The mean and standard deviation of their age, work experience in clinical setting, and ICUs were 31.43 ± 5.31, 2.74 ± 3.7, and 4.72 ± 3.67 years, respectively.

There were two groups of patients in the study, pre-interventional and interventional phase group. Data analysis before applying the CPOT showed that patients in two groups (pre-interventional and interventional phase) were matched and there was no significant difference in terms of their age (*p* = 0.937), gender (*p* = 0.996), and the cause of hospitalization (*p* = 0.997) (Table 2).

Also, comparison of the patients' physiological parameters (vital signs and mean arterial blood oxygen saturation) before implementing the CPOT did not show statistically significant difference among them (Table 3). This data showed no statistical difference between two groups in severity of pain at this phase.

However, after implementing the CPOT, there was a significant increase in total score of nursing practice in pain assessment per patient per day compared with before (7.2 ± 2.48 vs. 1.03 ± 1.63, *p* <0.001) and nurses used this tool to measure the severity of pain in three stages; before and after the treatment of pain and at peak effect time of analgesics, which were 1.6 ± 0.75, 5.5 ± 1.08, and 2.02 ± 0.48, respectively.

According to the results, there was a statistically significant difference (*p* = 0.018) between the mean amount of analgesic administration before (7.95 ± 8.77) and after implementing the CPOT (11.01 ± 11.04). In addition, the frequency of analgesic administration after intervention (4.16 ± 0.99) was significantly (*p* <0.001) more than before intervention (2.91 ± 1.38). (Table 4). However, there was no significant statistical difference in the type of administered analgesic before and after implementing the CPOT (*p* >0.05) (Table 5).

**Table 1:** Description of the critical-care pain observation tool (CPOT)

*Indicator*	*Description*	*Score*	
Facial expression	No muscle tension observed	Relaxed, neutral	0
	Presence of frowning, brow lowering, orbit tightening and levator contraction	Tense	1
	All of the above facial movements plus eyelid tightly closed	Grimacing	2
Body movements	Does not move at all (does not necessarily mean absence of pain)	Absence of movements	0
	Slow, cautious movements, touching or rubbing the pain site, seeking attention through movements	Protection	1
	Pulling tube, attempting to sit up, moving limbs/thrashing, not following commands, striking at staff, trying to climb out of bed	Restlessness	2
Muscle tension Evaluation by passive flexion and extension of upper extremities	No resistance to passive movements	Relaxed	0
	Resistance to passive movements	Tense, rigid	1
	Strong resistance to passive movements or incapacity to complete them	Very tense or rigid	2
Compliance with the ventilator (intubated patients)	Alarms not activated, easy ventilation	Tolerating ventilator or movement	0
	Alarms stop spontaneously	Coughing but tolerating	1
	Asynchrony: blocking ventilation, alarms frequently activated	Fighting ventilator	2
Or			
Vocalization (extubated patients)	Talking in normal tone or no sound	Talking in normal tone or no sound	0
	Sighing, moaning	Sighing, moaning	1
	Crying out, sobbing	Crying out, sobbing	2
Total, range	0-8		

**Table 2:** Patient characteristics (before-and-after the CPOT implementation)

*Variable/group*		*Post-Interventional group*	*Pre-interventional group*	*p value*
Age (mean ± SD)		48.65 ± 17.77	48.19 ± 14.14	0.937
Gender N (%)	Female	66 (55)	67 (55.8)	0.996
	Male	34(45%)	33 (44.2%)	
Cause of ICU admission N (%)	Gastric cancer	27 (22.5)	27 (22.5)	0.997
	Intra-cerebral hemorrhage	8 (6.8)	8 (6.8)	
	Lung cancer	17 (14.2)	18 (15)	
	Pulmonary infection	9 (7.5)	9 (7.5)	
	Subdural hemorrhage	11 (11.2)	11 (11.2)	
	Esophageal cancer	6 (5)	5 (4.2)	
	Multiple trauma	42 (35)	42 (35)	

**Table 3:** Patients' physiological parameters at baseline (before-and-after CPOT implementation)

*Physiological parameters*	*Pre-interventional group*	*Post-Interventional group*	*p value*
Heart rate	77.66 ± 11.57	77.66 ± 11.64	0.965
Respiratory rate	20.68 ± 11.4	18.88 ± 4.85	0.113
Systolic blood pressure	127.7 ± 14.29	127 ± 14.3	0.722
Diastolic blood pressure	71.82 ± 8.75	72.62 ± 9.30	0.493
Oxygen saturation (%)	97.05 ± 1.51	97.05 ± 1.51	0.995

**Table 4:** Dosage and frequency of administered analgesic before-and-after the CPOT implementation

*Administered analgesic*	*Pre-interventional group (M± SD)*	*Post-interventional group (M± SD)*	*p value*
Dosage (mg)	7.95±8.77	11.01±11.04	0.018
Frequency	2.91± 1.38	4.16±0.99	<0.001

**Table 5:** Analgesics prescribed before-and-after the CPOT implementation

*Analgesic*	*Pre-interventional group N (%)*	*Post-interventional group N (%)*	*p value*
Morphine	95 (79.2)	83 (69.2)	0.107
Pethidine	17 (14.2)	30 (25)	
Apotel	8 (6.7)	7 (5.8)	

## DISCUSSION

Based on the findings, implementing the CPOT can lead to a significant difference in the amount and frequency of administered analgesics. It means using CPOT had led to a more accurate assessment of pain among hospitalized patients in the ICU. Gallo et al. reported a 33% increase in pain assessment times after training nurses about the use of neonatal and infant pain scale.^22^

Findings of Hirsch et al. also indicated that the behavior pain scale (BPS) training resulted in up to 76.1% increase in pain assessment by nurses.^23^ Safari et al. showed that the use of BPS was effective in the ability to detect and monitor pain in patients with decreased level of consciousness, who are not able to express their pain. This enables the healthcare providers to diagnose and manage pain based on the behavioral symptoms. In addition, they concluded that the use of objective indicators by ICU nurses in the process of pain assessment is necessary.^24^

The comparison of the amount and frequency of analgesic administration during the first 24 hours of ICU admission showed a statistically significant difference before and after implementing the CPOT.

According to the results, the amount and frequency of analgesic administration after the intervention was significantly higher than before. These results are consistent with the results of Williams et al. which showed that the use of pain behavioral tool increases administration of analgesics in ICU patients. They claimed that this was due to increased ability of nurses to evaluate the pain.^25^ Similarly, Rose et al. reported the use of CPOT increases the administration of analgesics in the ICU.^26^ However, Gélinas et al. and Arbour et al. reported that use of CPOT decreases the use of analgesics and sedatives.^8,15^

It is thus clear that the use of objective tools leads to a more accurate estimation of pain intensity. This is of great importance since the type and amount of analgesics for each patient should be based on assessing the severity and cause of his/her pain.^19^

## CONCLUSION

Applying CPOT as an objective mean of pain assessment was effective in improving the performance of ICU nurses in assessment and management of patients' pain. It increased the amount and frequency of analgesic administration. We can recommend that COPT is a useful tool for assessment and management of pain in ICU patients and should be implemented in all ICUs.
